# Interlaboratory comparison of culture- and PCR-based methods for *Legionella pneumophila* detection in drinking water samples

**DOI:** 10.1128/aem.00236-25

**Published:** 2025-05-29

**Authors:** Maria Scaturro, Antonietta Girolamo, Antonino Bella, Maria Cristina Rota, Anna Maria Marella, Rossana Amari, Massimiliano Ingrassia, Daria Barberis, Chiara Romano, Francesca Borney, Florida Damasco, Maria Teresa Buratto, Monica Fortin, Gaetano Caricato, Giovanna La Vecchia, Filomena Casaburi, Melania Dragone, Mario Cavallaro, Giovanna Tozzi, Elisabetta Ceccarelli, Maria Anna Coniglio, Antonella Agodi, Valentina Coroneo, Adriana Sanna, Maria Luisa Cristina, Anna Maria Spagnolo, Sandra Cristino, Simona Spiteri, Laura De Lellis, Daniela De Mirto, Fabio Ferrari, Maria Giovanna Guiso, Marina Foti, Elisabetta Graziano, Marinella Franchi, Antonella Felice, Anna Giammanco, Teresa Fasciana, Annalisa Grucci, Elena Ballarini, Fabrizia Helfer, Ivan Corradi, Pasqualina Laganà, Alessio Facciolà, Antonella Mansi, Anna Maria Marcelloni, Isabella Marchesi, Annalisi Bargellini, Marianna Mari, Sabina Palmieri, Paola Miana, Stefano Della Sala, Osvalda De Giglio, Maria Teresa Montagna, Giusy Diella, Anna Moschin, Giorgia Lugarini, Claudio Ottaviano, Andrea Caprini, Anna Maria Rossi, Mariangela Pagano, Mariella Talini, Donella Gestri, Giovannella Vespa, Carla Croce, Mariagabriella Viggiani, Silvia Livi, Maria Luisa Ricci

**Affiliations:** 1 Department of Infectious Diseases, Istituto Superiore di Sanità 9289, Rome, Italy; 2 ESCMID Study Group for Legionella Infections (ESGLI) 454626 , Basel, Switzerland; 3 ARPA Reggio Emilia, Reggio Emilia, Italy; 4 Laboratorio di Sanità Pubblica ATS, Brescia, Italy; 5 ARPA Valle d’Aosta, Laboratorio di Microbiologia, Saint-Christophe, Italy; 6 ARPAV - Dipartimento Regionale Laboratori Servizio Analisi Biologiche Laboratorio EST, Venezia Mestre, Italy; 7 ARPA Basilicata, Laboratorio di Microbiologia, Matera, Italy; 8 ARPA Calabria, Laboratorio Bionaturalistico, Catanzaro, Italy; 9 ARPA Piemonte, Novara, Italy; 10 ARPA Umbria, Sez. Biomonitoraggio Acque – Ecotossicologia, Perugia, Italy; 11 Dipartimento di Scienze Mediche, Chirurgiche e Tecnologie Avanzate "G.F. Ingrassia", Università di Catania, Catania, Italy; 12 Laboratorio Regionale di Riferimento per la Diagnosi ambientale di Legionella. Dipartimento di Scienze Mediche e Sanità Pubblica, Università degli Studi di Cagliari 3111, Cagliari, Italy; 13 Dipartimento di Scienze della Salute, Università di Genova, Genova, Italy; 14 Dipartimento di Scienze Biologiche, Geologiche e Ambientali (BIGEA) Alma Mater Studiorum - Università di Bologna 9296, Bologna, Italy; 15 HERAtech s.r.l. -- Laboratori settore biologico, Bologna, Italy; 16 Sterimed, S.r.l., Milano, Italy; 17 Direzione Ricerca e Sviluppo, Centro Ricerche Gruppo CAP, Segrate, Italy; 18 Laboratorio di Prevenzione ASL, Milano, Italy; 19 ARPA FVG - SOC Laboratorio SOS, Laboratorio Alimenti e Microbiologia, Udine, Italy; 20 Laboratorio di Riferimento Regionale per la Sorveglianza ed il Controllo della Legionellosi. Dept. ProMiSe", Università degli Studi di Palermo 18998, Palermo, Italy; 21 ARPA Marche, Pesaro, Italy; 22 Laboratorio di riferimento Regionale per la sorveglianza ambientale Legionellosi, Trento, Italy; 23 Laboratorio Regionale di Riferimento per la Sorveglianza Clinica e Ambientale della Legionellosi. AOU "G. Martino", Università degli Studi di Messina 18980, Messina, Italy; 24 Dipartimento Medicina Epidemiologia, Igiene del Lavoro e Ambientale, INAIL, Monte Porzio Catone, Italy; 25 Dipartimento di Scienze Biomediche, Metaboliche e Neuroscienze, Sezione di Sanità Pubblica, Modena, Italy; 26 Servizio Ambiente e Salute, Dipartimento Prevenzione e Laboratori integrato, ARPA Lazio, Roma, Italy; 27 VERITAS Spa Laboratorio, Venezia, Italy; 28 Dipartimento Interdisciplinare di Medicina, Università degli Studi di Bari “Aldo Moro” 60272 , Bari, Italy; 29 Laboratorio Analisi Acquevenete SpA, Monselice, Italy; 30 ACEA ELABORI, Roma, Italy; 31 Area Analitica, Laboratorio di riferimento Regionale per la sorveglianza ambientale della legionellosi, Dipartimento di Salerno, Agenzia Regionale Protezione Ambiente, Salerno, Italy; 32 Laboratorio di Sanità Pubblica USL TC Centro di Riferimento della Regione Toscana per le Legionellosi, Firenze, Italy; 33 ARTA Abruzzo, Centro di Riferimento Regionale Legionella Distretto Provinciale, L'Aquila, Italy; 34 ARPAE Bologna, Bologna, Italy; Georgia Institute of Technology, Atlanta, Georgia, USA

**Keywords:** *Legionella*, drinking water testing, Legiolert, real-time PCR, Legionnaires disease, *Legionella pneumophila*, EU Directive 2020/2184, ISO 11731, GVPC, BCYE

## Abstract

The European Directive 2020/2184 concerning the quality of water for human consumption now includes *Legionella* among the pathogens for assessment in domestic water systems. It states that “for risk-based verification and to complement spread-plate culture methods, rapid culture methods, non-culture-based methods, and molecular-based methods may be used.” In this study, 33 laboratories across Italy analyzed a number of unique water samples ranging from 10 to 30 for the presence of *Legionella pneumophila*. All laboratories used the standard spread-plate culture method, 31 laboratories also performed the Legiolert rapid liquid culture method (IDEXX Laboratories), and 27 out of 33 performed the DI-Check *Legionella pneumophila* real-time PCR method (Diatheva). In all, 23 laboratories executed all three methods. Data generated from 817 samples were collected and statistically analyzed. The Legiolert method allowed analysis with a smaller sample volume (100 mL and 10 mL), compared to the standard culture method with which it was shown to be comparable with K agreement values of 0.785 and 0.840 in the two mentioned volumes, respectively. The standard real-time PCR method was more sensitive (93%) than the spread-plate culture method. Sensitivity values of 95.2% and 98.8% were also obtained by comparing two new real-time PCR procedures with the spread-plate culture method, tested to shorten the analysis, and the standard culture method. Finally, data obtained from the analysis of drinking water samples with the spread-plate culture method using both Buffered charcoal yeast extract and non-selective glycine vancomycin polymyxin cycloheximide media showed the greater capacity of the latter in the recovery of *Legionella* (*P* < 0.0001).

**IMPORTANCE:**

*Legionella* is a waterborne fastidious pathogen that occasionally infects humans and can cause a severe form of pneumonia, called Legionnaires’ disease (LD), which, if not identified in a timely manner, can progress to multiorgan failure. The trend for LD cases is steadily rising, and prevention and control of water system contamination is the only way to stop or prevent the spread of further cases. In Italy, since 2005, a network of regional reference laboratories for *Legionella* prevention has made an important contribution to *Legionella* surveillance. According to the European Directive 2020/2184, techniques already known can be used for *Legionella* detection in addition to the standard culture method. To ensure the reliability of the results and guarantee the correct risk evaluation, a comparison between the standard culture method and real-time PCR and the Legiolert rapid liquid culture method was required.

## INTRODUCTION

*Legionella* is an opportunistic premise plumbing pathogen (OPPP) responsible for Legionnaires’ disease (LD) and is acquired through the inhalation of water microdroplets from contaminated potable water distribution systems. LD cases, generally categorized as travel, community, or hospital acquired, were reported at an overall rate of 1.9 cases per 100,000 people in 2020 in the EU (European Union) and EEA (European Economic Area), with Italy, Spain, France, and Germany accounting for the highest number of cases (1). *Legionella pneumophila* (*L. pneumophila*) is the leading cause of OPPP-associated outbreaks in the United States (2), and the World Health Organization has stated that *Legionella* pose the highest health burden of all water-borne pathogens (3). The New European Directive 2020/2184 concerning the quality of water for human consumption includes *Legionella* among the parameters that should be controlled, and Member States (MS) should ensure that the parameters included in this directive are monitored (4). A risk-based evaluation approach has been advocated for monitoring *Legionella* in different settings, with special focus on specific buildings that have been named as priority premises. Based on this assessment, MS should take all necessary measures to ensure, among other things, that adequate management and control measures are put in place. Therefore, the water safety plan is a more effective approach to assess and minimize the risk of *Legionella* in water systems, which includes both its detection and quantification, which are essential factors for making decisions on water management (4).

With 3,111 cases and an incidence of 51.9 cases per million population in 2022, LD continues to increase in Italy, with 3,911 cases in 2023 and an incidence of 66.3 cases per million population (5). This underscores the critical need to manage *L. pneumophila* (5). The European Drinking Water Directive 2020/2184 states that for risk-based verification and to complement spread-plate culture, rapid culture methods, non-culture-based methods, and molecular-based methods may also be used.

The gold standard for detection and enumeration of *Legionella* in water samples is the spread-plate culture method, commonly performed according to ISO 11731:2017 (6). However, it is known that the spread-plate culture method is time-consuming, less sensitive for non-*pneumophila* species of *Legionella*, and is unable to detect *Legionella* that are within amoeba or in a viable but not culturable state (7–9). Given these and other limitations, this study was undertaken to assess the performance of validated commercial kits on Italian drinking water samples, which could offer practical advantages when used for risk-based verifications for *L. pneumophila*.

An alternative culture method is the Legiolert test (IDEXX Laboratories), which is based on a bacterial enzyme detection technology that highlights the presence of *L. pneumophila* through a change in the color of the medium. Several studies comparing this liquid culture method with traditional plate culture methods, including studies according to ISO 17994, have shown that Legiolert detects similar counts of *L. pneumophila* in potable water samples as the plate culture method. In 2021, US EPA researchers, Boczek et al., in a 185-sample comparison study, detected *L. pneumophila* in 83% and 85% of the samples with Legiolert and the plate culture method, respectively (10). Authors concluded that the two methods were statistically equivalent and that Legiolert showed a high degree of specificity of 96.5% (i.e., 3.5% false positives and 0% false negatives) versus the plate culture method. Scaturro et al. also demonstrated that no significant difference was found between either the Legiolert 10 mL and Legiolert 100 mL vs. the plate culture (*P* = 0.9 and *P* = 0.3, respectively) or between the Legiolert 10 mL vs. Legiolert 100 mL tests (*P* = 0.83; 11).

Spies et al. showed that Legiolert yielded, on average, higher counts of *L. pneumophila* than the ISO 11731–2 method, although the comparison with ISO 11731 was inconclusive due to the number of samples needing to be tested (12). In the same study, comparisons of Legiolert using 100 mL and the adoption of ISO 11731 or ISO 11731-2, as recommended by the Federal Environmental Agency (2012), did not show any conclusive difference, regardless of whether non-*pneumophila* species of *Legionella* were included in the evaluation. Legiolert has a high specificity for *L. pneumophila* of 97.9%, which compares favorably to the specificity of 95.3% quoted for ISO 11731. The authors concluded that Legiolert provides a significant improvement in the enumeration of *L. pneumophila* from drinking water and related samples (12).

Finally, Monteiro et al. showed that Legiolert, compared to data obtained with ISO 11731 and v-PCR for quantification of *L. pneumophila* in potable and non-potable waters, revealed concentrations of *L. pneumophila* greater than ISO 11731 and generally similar results to those of v-qPCR. The Legiolert method was highly specific and easy to use, representing a significant advancement in the quantification *of L. pneumophila* from potable and non-potable waters (13).

Other choices include well-established molecular-based techniques with excellent specificity and sensitivity, including the real-time PCR assay. To differentiate *L. pneumophila* from other species and *L. pneumophila* serogroup 1 from other serogroups, a number of real-time PCR assays have been proposed. Furthermore, intra-amoeba and viable but non-culturable *Legionella* were detected in water samples using real-time PCR assays (14–16).

Real-time PCR assays that have been validated according to ISO 12869:2019 are recommended over in-house ones to ensure that performance and quality control requirements are met, and that the assay is robust and accurate (17, 18).

The standard spread-plate culture method, the DI-Check *Legionella pneumophila* real-time PCR method (Diatheva), and the Legiolert liquid culture method (IDEXX) were used in a multicenter study to analyze potable water samples, to determine which techniques would best supplement the spread-plate culture method. Water samples from various sources, obtained from the laboratory’s routine operations, were examined for the study. In this study, the use of non-selective buffered charcoal yeast extract (BCYE) agar plates according to ISO 11731:2017 was also investigated. Furthermore, to enhance the application of real-time PCR for the detection of *Legionella* in environmental samples, alternative DNA extraction protocols utilizing reduced water volumes were evaluated.

## RESULTS

### Spread-plate culture method

Overall, 817 drinking water samples were analyzed by spread-plate culture on BCYE and GVPC media, and 54.6% (*n* = 446) were positive (Table 1). *L. pneumophila* was the prevalent species (88%), while only 12% (54 of the 446) of positive water samples included *Legionella* non*-pneumophila*. Of the 54 samples with other species, 33% also included *L. pneumophila*. Overall, 45.4% (*n* = 371) of the samples were negative on both types of media, and 34.8% (285) were positive on both, while 14.8% (*n* = 121) were positive only on GVPC and 4.9% (*n* = 40) were positive only on BCYE (Table 1), with a significant difference between the two (McNemar’s test, *P* < 0.0001). However, when the number of colony-forming units per L (CFU/L) of water sampled was determined and samples outside the appropriate range (<50 CFU/L) were excluded, there was no significant difference between the CFU/L on BCYE and GVPC agar plates (*P* = 0.2331) (Table 2). Based on CFU/L count, 40 samples were positive on BCYE and negative on GVPC, but 121 samples were positive on GVPC and negative on BCYE, and this difference was statistically significant (McNemar’s chi^2^ = 40.75) (Table 2). Non-*pneumophila* species of *Legionella* were detected in only 18 water samples: 38.8% (*n* = 7) on GVPC, 22.2% (*n* = 4) on BCYE, and the remaining 7 on both media. There were 45 samples that were uncountable on BCYE agar plates due to interfering microbial flora, while they were all positive on GVPC agar plates.

**TABLE 1:** Recovery of *Legionella* in *w*ater samples using BCYE and GVPC^*a*^

BCYE	GVPC		
	Negative (%)	Positive (%)	Total
Negative (%)	371 (45.4)	121 (14.8)	492
Positive (%)	40 (4.9)	285 (34.8)	325
Total	411	406	817

^*a*^ McNemar’s test *P*-value < 0.0001.

**TABLE 2:** Number of positive/negative water samples based on the range of both *Legionella pneumophila* and *Legionella* not-*pneumophila* CFU/L

	Range of CFU/L on GVPC				
	<50^*a*^	50–1,000	1,001–10,000	>10,000	Total positive
Range of CFU/L on BCYE					
<50^*a*^	371	78	30	13	121
50–1,000	21	60	10	0	70
1,001–10,000	17	21	86	9	116
>10,000	2	1	14	84	99
Total positive	40	82	110	93	285

^*a*^ Interpreted as a negative result (0 CFU/L of *Legionella pneumophila*) and not included in totals.

### Legiolert tests

**Fig 1:**
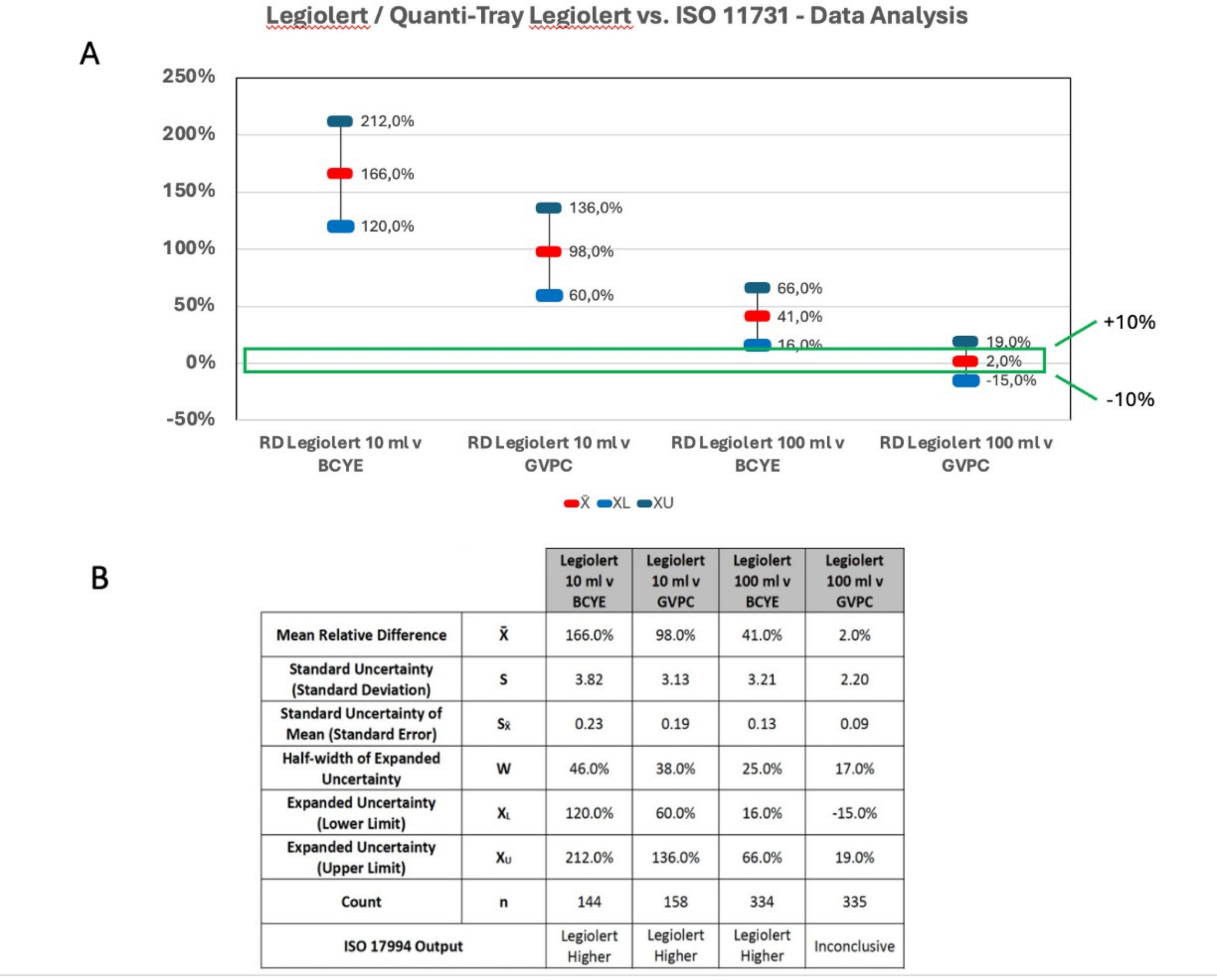
(**A**) Graphical representation of ISO17994 analysis of data generated during the trial. The mean relative difference for all comparisons is above “0.” In three out of four comparisons, the lower confidence limit was above the upper threshold (10%) recommended for drinking water, indicating that Legiolert is more sensitive. (**B**) Summary of ISO 17994 mean relative difference analysis comparing the Legiolert 100 mL (717 samples) and 10 mL (272 samples) tests versus the spread-plate method on BCYE and GVPC media (817 samples).

Of the 817 water samples analyzed by the spread-plate culture, many were also tested using the Legiolert 100 mL (717 samples) and Legiolert 10 mL (272 samples) tests. The Cohen’s Kappa coefficient (K value) calculated on samples analyzed by the Legiolert 100 mL test in comparison with samples analyzed by the spread-plate culture method was 0.785, indicating a good quality of agreement (*P* < 0.0001). A similar result was found for the 10 mL test. Likewise, a comparison of samples analyzed by both the Legiolert 100 mL test and the Legiolert 10 mL test was 0.840, also indicating very good agreement within these testing methods (Table 3). When compared according to the requirements in ISO 17994: 2014(19), the Legiolert 100 mL and 10 mL tests both showed greater sensitivity. The smaller number of samples required for the 10 mL comparisons to reach a conclusive output according to ISO 17994 is a function of the fact that in this study, the 10 mL method performed significantly differently than either BCYE or GVPC. In both cases, the 10 mL demonstrated to be more sensitive. In three out of four comparisons, the lower confidence limit was above the upper threshold (10%) recommended for drinking water, indicating that Legiolert is more sensitive than the spread-plate culture method on BCYE, but only the Legiolert 10 mL was more sensitive than the GVPC agar plates. When comparing the Legiolert 100 mL and GVPC agar plates, the results were consistent, with the two methods being equivalent, though a statistical analysis was inconclusive for the methods being different, indicating that more data are required for that comparison (Fig. 1).

**TABLE 3:** Legiolert tests versus spread-plate culture, percentage of sensitivity, specificity, positive and negative predictive values, agreement, and K value

Comparison	Sensitivity (95% CI)	Specificity (95% CI)	PPV^*a*^ (95% CI)	NPV^*b*^ (95% CI)	Agreement (%)	K value^*c*^
Legiolert 100 vs spread-plate culture	86.9 (84.4–89.4)	92.1 (90.1–94.1)	92.9 (91.0–94.7)	85.6 (83.0–88.1)	89.3	0.785
Legiolert 10 vs spread-plate culture	84.3 (80.0–88.6)	95.8 (93.4–98.2)	96.3 (94.0–98.5)	82.6 (78.1–87.1)	89.3	0.79
Legiolert 10 vs Legiolert_100	85.8 (81.7–90.0)	100	100	84.1 (79.7–88.4)	91.9	0.84

^*a*^ Positive predictive value.

^*b*^ Negative predictive value.

^*c*^ Cohen’s Kappa coefficient value.

### DI-Check *Legionella pneumophila* real-time PCR method

Of the 817 water samples that were analyzed by the spread-plate culture method, 599 were also tested with the DI-Check *Legionella pneumophila* real-time PCR method. 200 of the 599 were also used for DNA extraction using the Exp1 alternative protocol and 178 of the 599 with the Exp2 alternative protocol. The percent positive samples from the standard and two alternate protocols, Exp1 and Exp2, were 73.62%, 79% and 64%, respectively.

The K values for comparisons between the DI-Check *Legionella pneumophila* standard and Exp1 methods versus the spread-plate culture method were 0.5 and 0.48, respectively, consistent with moderate agreement. There was slightly better agreement between the Exp2 protocol and the spread-plate culture method (K = 0.66) (Table 4). When the different methods of DNA extraction were compared with each other, there was substantial agreement. The K values for Exp1 and Exp2 compared with the standard protocol were 0.63 and 0.85, respectively (Table 5).

**TABLE 4:** Comparison of the DI-Check *Legionella pneumophila* real-time PCR test, standard and alternate experimental methods, with the spread-plate culture method

Comparison	Sensitivity (95% CI)	Specificity (95% CI)	PPV (95% CI)	NPV (95% CI)	Agreement (%)	K value
Standard method vs spread-plate culture	93.9% (92–95.8)	53.9% (49.9–57.9)	73.5% (69.9–7)	86.7% (83.9–89.4)	77.0	0.50
Exp1 vs spread-plate culture	95.2% (92.2–98.2)	48.7% (41.7–55.5)	76.0% (70.0–81.8)	85.7% (80.8–90.5)	78	0.48
Exp2 vs spread-plate culture	98.8% (97.2–100.4)	67.7% (60.8–74.6)	73.7% (67.2–80.1)	98.4% (96.6–100.2)	82.6	0.66

**TABLE 5:** Comparative analyses of DI-Check *Legionella pneumophila* standard, Exp1, and Exp2 protocols

Comparison	Sensitivity (95% CI)	Specificity (95% CI)	PPV (95% CI)	NPV (95% CI)	Agreement (%)	K value
Exp1 vs DI-Check *Legionella pneumophila* standard method	92.4% (88.7–96.0)	69.8% (63.4–76.1)	91.8% (88.0–95.6)	71.4% (65.2–77.7)	87.5	0.63
Exp2 vs DI-Check *Legionella pneumophila* standard method	91.8% (87.8–95.8)	96.4% (93.7–99.2)	98.2% (96.3–100.2)	84.4% (79.0–89.7)	93.3	0.85

## DISCUSSION

In this broad study involving 33 independent Italian laboratories, two effective methods for detection and enumeration of *Legionella pneumophila*, one based on liquid culture and one based on real-time PCR, were compared with the standard spread-plate culture method according to ISO 11731:2017. Both methods tested, Legiolert and DI-Check *Legionella pneumophila*, were found to be good candidates to complement the spread-plate culture method in risk assessment and to monitor *Legionella*, as required by the new European Directive, Annex III part A (4). This new directive, concerning the quality of water for human consumption, highlights the importance of monitoring the genus *Legionella* using ISO 11731 as the reference method. However, much of the literature reports that *L. pneumophila* is the predominant species responsible for environmental spread in Europe, and particularly in Italy, which has one of the highest incidences of LD (52 cases per million inhabitants in 2022) and where *L. pneumophila* is responsible for 100% of diagnosed cases (1, 5). For this reason, even though we are aware that surveillance data may be biased due to the frequent use of clinical diagnostic methods that specifically detect *L. pneumophila* SG1, this study focused on *L. pneumophila*. A primary goal of this study was to identify more sensitive testing methods to complement the spread-plate culture method, even if the presence and frequency of other *Legionella* species identified by the latter method have been noted.

As already demonstrated (10–13), Legiolert is an effective method to detect and enumerate *L. pneumophila* in potable water samples when compared to the spread-plate culture method. This study confirmed these findings across a large cross-section of settings, in 31 independent laboratories from 22 regions across Italy, and helps answer the question of the feasibility of the method being implemented as an alternative method at a national scale. None of the 31 laboratories reported difficulty running the method or interpreting the results from the Legiolert Quanti-trays. The largest number of samples (717) was analyzed with the Legiolert 100 mL protocol, which offers a limit of detection consistent with the threshold limits articulated in the European Directive 2020/2184 (4). Per ISO 17994: 2014 analysis, the 100 mL Legiolert protocol showed significantly higher sensitivity compared to BCYE spread-plate results and equivalent performance to GVPC spread-plate culture results. This means that the Legiolert tests not only complement but can also be an effective substitute for the spread-plate culture method when the detection of *L. pneumophila* alone is appropriate.

In this study, a lower mean relative difference (2%) between 100 mL Legiolert protocol and the GVPC culture ISO 11731 results (335 paired counts) was calculated compared with the mean relative difference (35.3%) determined by Sartory et al. in a previous study, involving four laboratories and analyzing a comparable number of samples (290 paired counts) (20). The laboratories in this study found an unbiased variance with greater or lesser sensitivity between the two technologies, in contrast to the Sartory et al. study, where higher counts were determined with Legiolert (12.4%–73.1% mean relative difference). In this study, the participating laboratories analyzed the samples according to the ISO-11731:2017, adopting filtration (1L) and washing procedures specific for each laboratory but always compliant with the ISO norm. The four laboratories in Sartory et al. adhered to a specific, consistent protocol based on ISO-11731-2 (membrane filtration followed by acid washing and plating on GVPC) using the same volume of Legiolert test (100 mL). Perhaps the largest difference observed between Legiolert and the spread culture method in the Sartory et al. study could be due to the use of the same volume of water in the two methods, which could have disadvantaged the spread culture method that usually uses larger volumes. In our study, all laboratories used 1 L of water for comparing Legiolert, probably increasing the sensitivity of the culture method and showing smaller differences between the two methods in the statistical analyses. These results confirm that Legiolert can be widely adopted as an alternative method, having also demonstrated its validity between different laboratories. The agreement between the Legiolert 100 mL and Legiolert 10 mL tests, as determined by Cohen’s kappa coefficient (0.84), points to a more streamlined workflow and similarly effective results with a smaller volume sample. Albeit over a smaller number of samples (*n* = 158), the Legiolert 10 mL test also demonstrated agreement with the BCYE and GVPC spread-plate methods, with higher sensitivity than both of these methods. One unexpected finding of the study was that the average mean relative difference for the subset of laboratories (*n* = 11) that generated results with the Legiolert 10 mL test was 98% (Legiolert higher) relative to the GVPC test results, which was notably larger than the 2% average greater sensitivity relative to GVPC seen by the 31 laboratories running the 100 mL protocol. This has not been seen previously in other studies or by the method developer, so additional information about the process and results from using the Legiolert 10 mL protocol was requested from the six out of ten laboratories that saw these higher counts for the 10 mL protocol. No patterns emerged from this further investigation, so we plan to explore this finding further in an upcoming study.

DI-Check *Legionella pneumophila* is a real-time PCR-based method validated by AFNOR for the detection and quantification of *L. pneumophila* in all types of water samples, according to NF T90-471 and ISO 12869 (17). The DI-Check *Legionella pneumophila* method performed very well. It is highly sensitive and has an elevated negative predictive value when compared to the spread-plate method. It was, as expected, moderately specific (53%), due to the ability of real-time PCR to detect a higher number of positive samples (Table 4). There are widely discussed points concerning the overestimation of positive samples using this method versus the spread-plate culture method, and concerning the different measurements used by the two methods, which are Genomic Unit /L (GU/L) and CFU/L (14, 16, 21). GU/L calculations are independent of cell viability and can therefore be affected by the presence of dead cells, a fraction of which may result from the disinfection treatments used in potable water systems. In this study, 70% (*n* = 419) of the samples analyzed by DI-Check *Legionella pneumophila* were from water systems that did not use disinfecting treatments, and among the 117 samples that were positive by real-time PCR but negative by spread-plate culture, 29% (*n* = 34) contained interfering microorganisms that could have masked the presence of *Legionella* in spread-plate cultures. This suggests that most probably the exceeding number of positive samples by the DI-Check *Legionella pneumophila* method were true-positive results that escaped notice in the spread-plate culture method.

According to the Italian national guidelines for *Legionella* prevention and control, real-time PCR may be used as a method for rapid screening for the presence of *Legionella* in water samples (21). Indeed, thanks to the high negative predictive value of real-time PCR, negative samples can be reported without spread-plate culture confirmation (7, 16, 21). On the contrary, samples testing positive by real-time PCR must subsequently be analyzed by spread-plate culture to determine the *Legionella* concentration levels. Therefore, in Italy, laboratories intending to use real-time PCR for *Legionella* detection must collect at least 2 L of water, and very often must additionally perform the spread-plate culture method. For this reason, the real-time PCR method is seldom used. To evaluate possibilities for reducing the time and effort required for laboratory analyses, this study explored two alternative experimental procedures, Exp1 and Exp2, for preparing samples for real-time PCR, both utilizing a portion of the 1 L water sample concentrated for culturing purposes (equivalent to 500 mL of water sample) rather than starting with an additional 1 L water sample as for the standard method. They differed in the way samples were prepared for DNA extraction: for Exp1, the filter was placed into 2 mL lysis buffer, heated, and the DNA was purified using a DNApure Water Isolation kit; for Exp2, the filter was placed into 500 µL of lysis buffer, heated, and then the suspended DNA was used directly in the real-time PCRs (Fig. S1). A comparison of the spread-plate culture method to any of the three real-time PCR protocols showed similar results. When the two alternate experimental protocols were compared with the standard protocol, Exp2 showed very good agreement and high sensitivity, specificity, PPV, and NPV (Table 5). This may be explained by the fact that there was less sample manipulation since DNA was isolated from all cells embedded on the filter membrane, minimizing overall loss. In addition, considering the very good K value of Exp2 in comparison with the standard protocol, Exp2 appears to be more effective at detecting *Legionella* DNA (Table 5). Further testing of the Exp1 and Exp2 protocols should be carried out on a higher number of water samples to confirm that this is consistently advantageous before introducing either of these experimental procedures into routine use in testing labs.

In this multicenter study, the detection and enumeration of *Legionella* by the spread-plate culture method on both selective GVPC and non-selective BCYE media, according to ISO 11731, were compared. Our current results are consistent with what we reported previously (11), in that there was a statistically significant higher number of positive samples on GVPC compared to BCYE agar plates, which we attribute, at least in part, to there being many BCYE plates that were unreadable due to a high level of interfering growth.

The current data confirm and strengthen our previous data in many respects, contesting statements in Ditommaso et al. that attributed our previous results to a poor ability to recognize *Legionella* colonies, differing bench protocols, and differing statistical analyses (22, 23). Twenty-one laboratories participating in this study have been accredited according to ISO 11731 by Accredia, the Italian accreditation body, meaning that those carrying out the work are trained in microbiology and are skilled at analyzing the growth of *Legionella*. Furthermore, it is unlikely that different bench protocols may have affected the results because, in the current study, the work from 33 different laboratories was comparable. Ditommaso et al. (23) reported a higher number of positive samples; however, their data come from a single laboratory, whereas our results are more robust due to the added value of interlaboratory variability. The different results between this study and the Ditommaso et al. (23) study could reflect a differing risk of interfering growth on the non-selective BCYE media. As stated above, our study included many unreadable BCYE plates, and this interference makes this medium less useful.

The usefulness of non-selective BCYE is that species of *Legionella* other than *pneumophila* do not grow well on selective media. However, in our analyses, non-*pneumophila* were seldom detected (number of samples with only L. non-*pneumophila* detected = 18), and only four were detected on BCYE agar plates, meaning that a measure of these species is of less importance.

In this study, we had a high number of positive samples (*n* = 446 of 817, 54.5%) and *L. pneumophila* was the predominant species (392 samples, 88%), with non-*pneumophila* species comprising only 12% (*n* = 54), which agrees with data reported in the literature (24–29). Our data are also representative of the frequency typically observed for *Legionella* species throughout Italy, given that the water samples analyzed in this study were collected from domestic water systems across the 21 regions where *Legionella* species likely vary, as they have been reported to have been in water samples taken across Spain (30).

The lack of any significant difference in the average CFU/L values in BCYE agar plates versus GVPC suggests that perhaps the additional effort of including BCYE media in the analysis is not worth the time, the additional personnel, or the generation of excess waste. In addition, other *Legionella* species are less pathogenic than *L. pneumophila*, as demonstrated by the low incidence of infection and the lack of cluster and/or outbreak events due to non*-pneumophila* species (1). The only exception is clusters of *Legionella longbeacheae* associated with potting compost (31–33).

A disadvantage of this study could be that different samples were examined using conventional techniques in several labs, some of which differed somewhat from one another. However, a specific laboratory’s standard procedures were not the subject of the comparison. The study’s objective was to assess how well the results produced by several labs using their normal procedures agreed with one another. The robustness of the studied methodologies is demonstrated by the fact that the results were consistent across the different laboratories.

### Conclusions

In conclusion, we show that both Legiolert and the DI-Check *Legionella pneumophila* method are reliable methods that complement the spread-plate culture method for the detection of *Legionella* in water samples intended for human consumption. The use of Legiolert would shorten both the time and sample volume needed for analysis. Furthermore, the DI-Check *Legionella pneumophila* method provides the benefit of a rapid turnaround time and high sensitivity, which could further reduce the time to results as well as reduce the number of spread-plate analyses that are needed, if spread-plate cultures are carried out only when RT-PCR results are positive. In addition, we show that, using either Exp1 or Exp2 methods, the same concentrated water sample can be used for both RT-PCR and spread-plate cultures, which saves time and sampling volume.

The most recent European Directive 2020/2184 (4) concerning the quality of water for human consumption allows for the use of alternative methods to complement the spread-plate culture method. As a result, there should be a considerable increase in the amount of data that can be collected, and in the future, this should facilitate approval of these methods as reliable and accurate for *Legionella* determination across Europe*,* highlighting the importance of this study beyond Italy.

The joint action of adopting the new drinking water directive, implemented in Italy by a new law (30), and having experienced alternative rapid methods through the entire network of Italian laboratories will ultimately allow for better prevention and much more effective control of LD, which will be closely monitored in the future.

## MATERIALS AND METHODS

### Participating laboratories and water sampling

Thirty-three Italian laboratories participated in the study, including the National Reference Laboratory and 22 Regional Reference Laboratories for *Legionella*, as well as universities and private companies (Table 6). The laboratories were selected based on their qualifications for *Legionella* testing, according to ISO 11731:2017 by Accredia, the Italian accreditation body, and/or their participation in external quality assessment schemes for *Legionella* in water (Tables S1 and S2; 6). Each laboratory was asked to analyze the presence of *L. pneumophila* in a number of potable water samples ranging between 10 and 30, according to their daily routine, collecting samples in a single sterile bottle (containing a blocking solution for the disinfecting agent) at a volume sufficient to run all the methods they chose, which generally amounted to 3 L. All the laboratories were invited to run water samples by spread-plate culture, 31 performed the Legiolert 100 mL test, and 11 out of 31 also performed the Legiolert 10 mL test. Twenty-six of the laboratories also used real-time PCR: 4 of which used the slightly modified Exp1 protocol and 5 of which used the Exp2 protocol, which differs more significantly in that it excludes the DNA purification steps.

**TABLE 6:** *Legionella* testing methods compared in this study

Method	No. of laboratories performing each method	Variations	Additional details
Spread plate	33	Non-selective BYCE media	ISO 11731:2017 protocol (with lab-specific variations based on Annex J)
		Selective GVPC media	ISO 11731:2017 protocol (with lab-specific variations based on Annex J)
Legiolert liquid culture method (IDEXX)	11	10 mL sample method	According to the manufacturer’s protocol and ASTM 8429:2021
	31	100 mL sample method	According to the manufacturer’s protocol and ASTM 8429:2021 Includes an adjustment for water hardness
DI-Check *Legionella pneumophila* method (Diatheva)	26	Standard method:	According to the manufacturer’s protocol and ISO12869: 2019 Briefly: 1 L of water; filtered, heated, and DNA isolated using the DNApure water isolation kit.
	4	“Exp1”	Slight modification of the standard method. Briefly: Previously concentrated sample (equivalent of ~500 mL) was filtered a second time, heated, and DNA purified using the DNApure water isolation kit.
	5	“Exp2”	A major modification of the standard method. Briefly: Previously concentrated sample (equivalent of ~500 mL) was filtered a second time, heated, and used directly in PCR without a purification step.

The sampled buildings were quite varied and mainly included hospitals, healthcare homes, and hotels but also included private homes, public offices, barracks, gyms, and school buildings (34). Generally, the water from health facilities and hotels was disinfected through building-level water treatment, while that from private buildings was not.

Many of the laboratories were already experienced in the alternative methods being assessed, and all other participants were trained in the methods to be performed. Laboratories reported the identity of the sampled buildings, the specific points of sampling, the temperature of the water samples, and the presence of any disinfectant treatments in the building.

### Spread-plate culture method

Water samples (1 L) were analyzed by each laboratory using the spread-plate culture method according to ISO 11731:2017, following the Decision Matrix in Annex J. The water samples were classified as matrix A, meaning with low interfering flora, and the filtration with the membrane washing procedure was utilized. Laboratories used their own standard detailed protocols to remove *Legionella* from the membrane (scraping, rubbing, etc.) and to concentrate the volume to 10 mL. Three milliliters of the concentrated sample was divided into three aliquots of 1 mL and treated as follows: no treatment, incubation at 50°C for 30 minutes, and treatment with an acid solution (6). Each aliquot was then seeded at 100 or 200 µL on non-selective *Legionella* BCYE (buffered charcoal yeast extract, ThermoFisher, United Kingdom) agar plates and on selective *Legionella* GVPC (glycine vancomycin polymyxin cycloheximide, ThermoFisher, United Kingdom) agar plates. Plates were inspected after 2, 3, 4, or 5 days and at the end of the incubation period (10 days). The remaining bacterial concentrate was used to perform additional tests described in Section 2.4. Per standard procedure, the highest counts for each sample were recorded and used for the comparison with the other methods.

At least three colonies were spread-plate cultured onto BCYE and BCYE without cysteine if only one morphological type was present. Otherwise, at least one representative of each type was cultured, and plates were incubated at 36°C ± 2°C for at least 48 hours. Colonies were identified by latex agglutination (ThermoFisher, United Kingdom), an immunochromatographic test (Vircell, Spain), or MALDI-TOF MS (Bio-Merieux, France).

### Legiolert test

The Legiolert test (IDEXX Laboratories, USA; (35), which is validated by AFNOR, was performed using 100 mL and/or 10 mL of each water sample, according to the manufacturer’s instructions. Briefly, for a Legiolert test using a 100 mL water sample, water hardness was measured, and depending on this determination, 0.33 mL or 1 mL of the supplied supplement kit was added. Then, the Legiolert blister pack content was added to the 100 mL of water sample, shaken, and poured into a Quanti-Tray/Legiolert. The Quanti-Tray was immediately sealed and incubated at 39°C for 7 days. For a Legiolert test using 10 mL, the sample volume was added to 90 mL of sterile water, and no hardness measure was performed. All the other steps were as for the 100 mL Legiolert test. Thirty-one laboratories performed the Legiolert test using 100 mL water samples, and 11 of them also performed the test on 10 mL water samples. Quanti-Trays from samples which were positive for Legiolert and negative by spread-plate culture were verified by subculturing an aliquot of Legiolert broth onto BCYE agar plates.

### DI-Check *Legionella pneumophila* real-time PCR

Real-time PCR experiments were performed using the DI-Check *Legionella pneumophila* kit (Diatheva, Italy), according to the manufacturer’s instructions, including the use of the DNApure Water Isolation kit (Diatheva, Italy) for DNA extractions following the procedure validated by AFNOR for reliability (36). As the DI-Check *Legionella pneumophila* kit is validated for several real-time PCR instruments, each laboratory used its platforms. Specifically, 17 labs used the CFX96 Real-Time Detection System and one the Chromo4 system (both from Bio-Rad Laboratories, USA). Four labs used the QuantStudio 5 and one the StepOne Real-Time PCR System (both from Applied Bio-System, USA). Lastly, two labs used the Rotor Gene Q (QIAGEN, Germany) and one the Agilent Stratagene Mx3000P (Agilent Technologies, USA). The manufacturer’s instructions for the DI-Check *Legionella pneumophila* method include concentration of water samples via filtration. Here, two additional procedures for DNA extraction, designated Exp1 and Exp2, were utilized. These procedures were included to explore the possibility of reducing the sample volume requirements for the method, such that the collection of a single 1 L sample would be sufficient to perform both the spread-plate culture and real-time PCR methods, rather than requiring 1 L of sample for each method. For the standard DNA extraction protocol, 1 L of a water sample is filtered, then the filter is placed in a tube containing 2 mL of the lysis buffer provided with the DNApure Water Isolation kit. After boiling, the DNA freed in solution is purified by column centrifugation. For the two alternate procedures, 5 mL of the 10 mL bacterial concentrate described in Section 2.2, which was prepared during the spread-plate culture analysis, was filtered a second time through a polycarbonate filter with a nominal porosity of 0.45 µm. For Exp1, the membrane filter was placed in a tube containing 2 mL of lysis buffer, heated at 95°C for 15 minutes, and then the DNA extraction proceeded according to the manufacturer’s instructions for the DNApure Water Isolation kit. For Exp2, the membrane filter was placed in 500 µL of sterile distilled water, heated at 95°C for 10 minutes, and the DNA freed in solution was analyzed by real-time PCR without any further purification. Finally, 5 µL of DNA from each extraction method was used in a real-time PCR (Fig. S1).

### Statistical analysis

Data from all contributing laboratories were analyzed for statistical significance using Stata software version 11.2 (Stata Corp, College Station, TX, USA). McNemar’s test was used to compare frequencies in paired data. The concordance between media was evaluated using the Cohen’s Kappa coefficient for which K < 0.20 = “poor,” K = 0.20–0.40 = “fair,” K = 0.40–0.60 = “moderate,” K = 0.60–0.80 = “good,” and K = 0.80–1.00 = “very good” (36). Specificity and sensitivity, as well as positive and negative predictive values (PPV and NPV, respectively), and 95% confidence intervals (CI) on both media were also calculated. To compare the Legiolert 10 mL and Legiolert 100 mL tests to spread-plate culture methods, mean relative difference analysis was performed according to ISO 17994:2014 (37). According to the ISO 17994 two-sided evaluation criteria, when the Upper (U) and Lower (L) limits of the confidence interval around the mean relative difference were respectively −10% ≤ xL ≤ 0 and 0 ≤ xU ≤ + 10%, the methods are determined to be “not different” (10% is the threshold value recommended for drinking water). Alternatively, if xL >0 or xU <0, the methods were determined to be “different.” Furthermore, the smaller the amplitude of this confidence interval, the greater the confidence in the statistical output.

## SUPPLEMENTAL MATERIAL

Fig. S1 aem.00236-25-s0001.pdf Graphical representation of DNA extraction procedure.

Table S1 aem.00236-25-s0002.xlsx Participating laboratories, samples analyzed, and methods performed.

Table S2 aem.00236-25-s0003.xlsx Raw results of spread-plate culture, Legiolert, and DI-Check Legionella pneumophila methods.

ASM does not own the copyrights to Supplemental Material that may be linked to, or accessed through, an article. The authors have granted ASM a non-exclusive, world-wide license to publish the Supplemental Material files. Please contact the corresponding author directly for reuse.
