# Supplementary material for: Interlaboratory comparison of culture- and PCR-based methods for Legionella pneumophila detection in drinking water samples
Source: Appl Environ Microbiol. 2025 May 29;91(6):e00236-25. doi: 10.1128/aem.00236-25 (PMC12175499; doi:10.1128/aem.00236-25)
Supplement: Fig. S1 — Graphical representation of DNA extraction procedure. [file aem.00236-25-s0001.pdf]

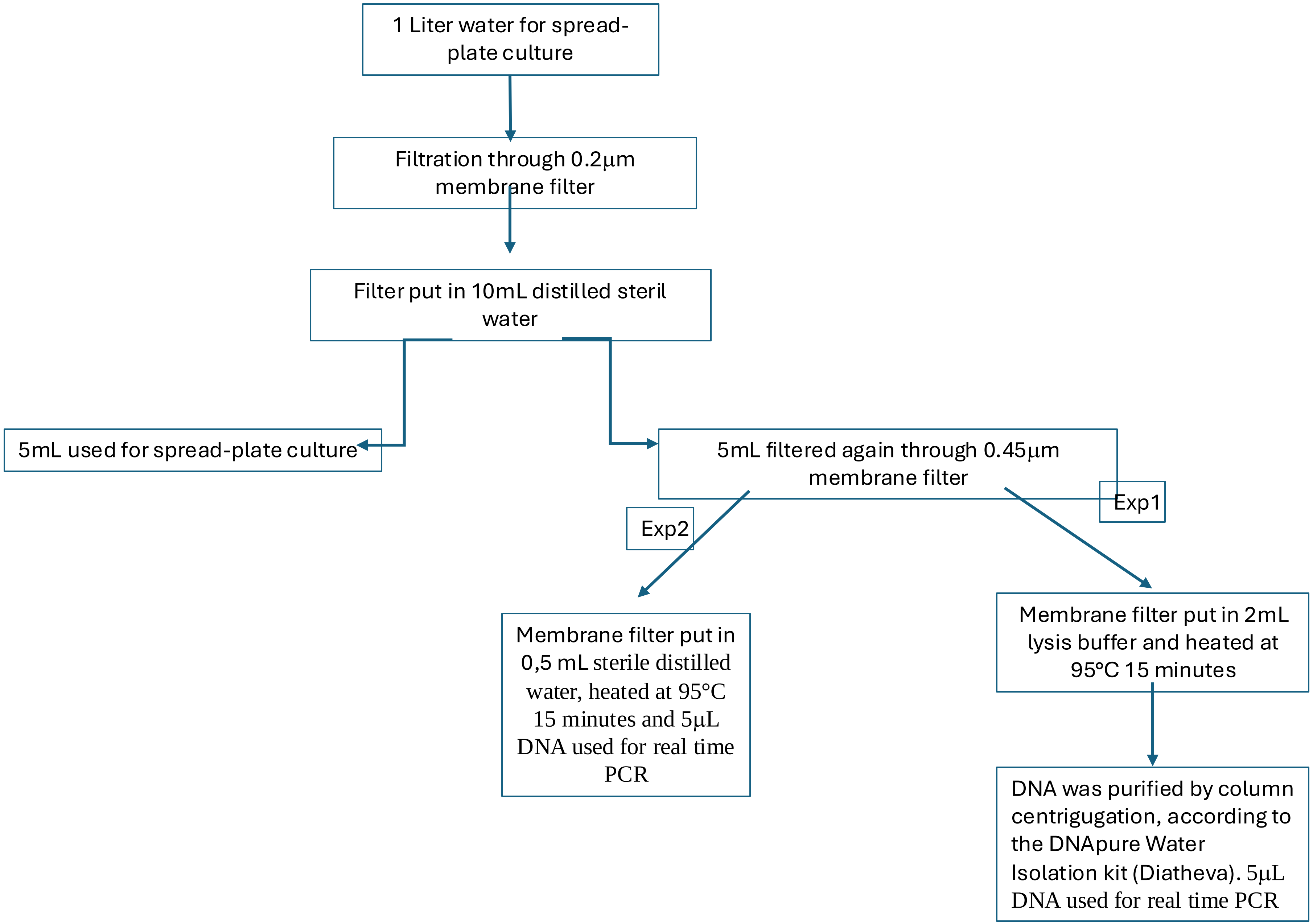

Figure S1. Graphical representation of the two procedures used in addition to the standard one to extract DNA for subsequent Real Time PCR analysis.
